# Perceptual Surgical Knife with Wavelet Denoising

**DOI:** 10.3390/mi9020079

**Published:** 2018-02-13

**Authors:** Tao Li, Yuta Sunami, Sheng Zhang

**Affiliations:** 1 Institute of Innovative Science and Technology, Tokai University, Hiratsuka-shi 259-1292, Japan; taoli321@yahoo.com; 2 Micro/Nano Technology Center, Tokai University, Hiratsuka-shi 259-1292, Japan; 3 Department of Mechanical Engineering, Tokai University, Hiratsuka-shi 259-1292, Japan

**Keywords:** surgical knife, field-effect transistor (FET), wavelet denoising, perception, robotic surgery

## Abstract

Robotic surgery is a new technology in medical applications and has been undergoing rapid development. The surgical knife, essential for robotic surgery, has the ability to determine the success of an operation. In this paper, on the basis of the principle of field-effect transistors (FETs), a perceptual surgical knife is proposed to detect the electrons or electric field of the human body with distinguishable signals. In addition, it is difficult to discriminate between the motions of surgical knives from the perceptual signals that are disturbed by high-frequency Gaussian white noise. Therefore, the wavelet denoising approach is chosen to reduce the high-frequency noise. The proposed perceptual surgical knife with the wavelet denoising method has the characteristics of high sensitivity, low cost, and good repeatability.

## 1. Introduction

The advent of robotic surgery has promoted the development of the tool–tissue interaction technique [1]. The critical component of a surgical robot is the perceptual sensor that provides different surgery information about the tissues. The most significant challenge is how to conduct an appropriate surgical treatment of small or tiny tissues under complex conditions. Taking vascular surgery as an example, it is difficult for the operator to determine whether the surgical knife or scalpel touches the blood vessels. In this case, any improper contact with the blood vessel will lead to intraoperative or postoperative hemorrhage [2].

To reduce the risk of operational failure, a camera platform with a three-dimensional video monitoring system has been developed in the field of telerobotic surgery [3]. However, the video-based robotic surgery system lacks haptic sensation (tactile or vibration) [4]. To overcome the above problem, the haptic interface has been proposed to provide a bidirectional (two-way) interaction between the human beings and the virtual environment [5]. Some tool–tissue interaction interfaces have been studied by using scissors, surgical knives, syringes, and so forth with the goal of haptic sensation. Liu et al. has proposed a force-sensing bipolar forceps with strain gauge sensors [6]. The minimum sensing force with the bipolar forceps ranges from 0.1 to 0.41 N. In addition, another bipolar forceps was designed with an accelerometer and position tracker to investigate the smoothness and dexterity of hand motions during surgical skill assessment. The normalized jerk index serves as the indicator of motion smoothness and dexterity [7]. The multi-sensory mechatronic device has been designed to locate the tumors with the capacitive tactile array, ultrasound transducer, and electric position tracker. The experimental results show that the tumor can be located with a force of 5–6 N in both the tactile and ultrasound modes [8,9]. The drawback is that the aforementioned tools are expensive and sensitive to external disturbances. Xie et al. have proposed an optical tactile array probe head that is capable of detecting tissue abnormality through the tactile force feedback (0 to 0.5 N with a resolution of 0.05 N). The probe head is comprised of two plastic optical bundles with 16 individual optical fibers in each bundle [10]. However, the light intensity propagated in the optical fiber is susceptible to mechanical noise.

The field-effect transistor (FET)-based tactile sensors characterized by a high sensitivity, a fast response time, miniaturization, and a low cost have considerable potential for the design of a perceptual surgical knife [11]. The FET sensors use the electric field to control the device’s behavior. Different FET sensors have been proposed with distinguished materials, for example, ZnO nanorods with a high sensitivity of 1.6 mA/(μM·cm${}^{2}$) for continuous glucose monitoring [12], enzyme-catalyzing-based FET sensors with a sensitivity of 10 mM and Nernstian response of 59.2 mV/decade [13], and ionic liquids with a pressure sensitivity of $2.2\times 10^{3}$ kPa${}^{-1}$ [14]. Currently, FET-based bio-inspired electronic skins are being developed in the literature, such as nanowire FET-based robotic tactile sensing skins [15], and graphene tribotronic transistor-based artificial skins [16]. The purpose of this work is to design a perceptual surgical knife to detect the electrons or electric field in the human body by using the principle of FETs.

## 2. Fundamentals of Perceptual Surgical Knife

### 2.1. Sensing Principle of Perceptual Surgical Knife

**Figure 1:**
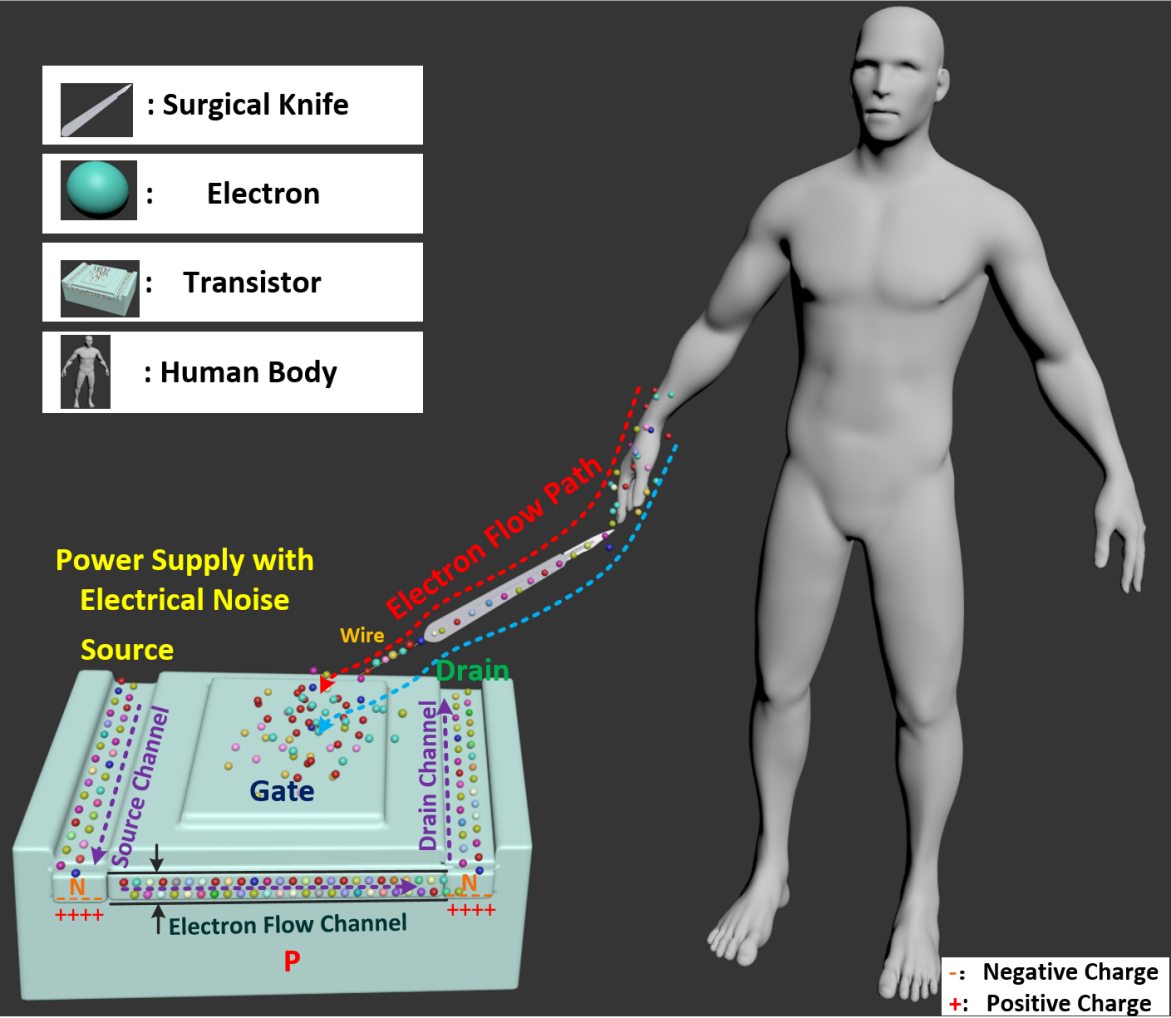
Sensing principle of the perceptual surgical knife. The electron flow channel inside the sensing transistor is expanded when the surgical knife touches the human body. The physical surgical knife serves as a medium for the electrons flowing from the human body to the sensing transistor. The electrons from the human body (moving along electron flow path) are used to control the conductivity of the electron flow channel, and the electrons from the power supply flow from the source to drain side (along the purple dashed line).

Figure 1 shows the schematic diagram of the proposed perceptual surgical knife consisting of a FET sensing transistor and a physical surgical knife. The FET transistor has three terminals: the source, drain and gate. As shown in Figure 1, the electrons from the power supply with electrical noise flow in the source channel. Through the electronic flow channel, finally, the electrons flow out of the drain channel. The gate terminal controls the conductivity of the electron flow channel by using an electric field. Unlike the traditional transistor powered by direct current (DC), the transistor of the proposed surgical knife is powered by DC with stochastic electrical noise. The boundary between the p−n junction forms a depletion region that prevents the electrons to flow from the source to the drain side. However, the channel is activated by the electric field generated by the electrical noise of the power supply. Therefore, the electric field created by the fluctuation of electrical noise is used to open the electron flow channel between two *N* junctions in the FET (refer to Figure 1). In addition, the voltage generated by the moving of electrons is amplified by the amplifier inside the surgical knife. Therefore, the voltages are detected at the output of the surgical knife without any inputs. The electron flow channel is expanded when the surgical knife obtains more electrons that generate the electric field to open the gate. The electrical impulse of the human body, formed by the imbalance between potassium and sodium ions inside and outside the cell, expands the electron flow channel; correspondingly, the voltage variation is detected at the output of the surgical knife. Hence, the motions of the surgical knife (tapping, sliding, and cutting) are detected because the electrons of the human body expand the electron flow channel of the FET transistor when the surgical knife touches the human body. The electric field, generated by the electrons of the human body, is given by the following equations [17]:

(1) $$E_{field}=\frac{q_{e}}{4\pi \varepsilon_{0}\kappa_{e}r^{2}}$$

where $E_{field}$ (unit: V/m) and $M_{field}$ (unit: tesla, T) are the electric and magnetic field, respectively; $q_{e}=1.6\times 10^{-19}\;$*coulombs* is the electron charge; $\varepsilon_{0}=8.85\times 10^{-12}$ F/m is the electrical permittivity constant; $\kappa_{e}=1$ is the dielectric constant; *r* is the distance between electrons.

### 2.2. Measurement Setup of Perceptual Surgical Knife

**Figure 2:**
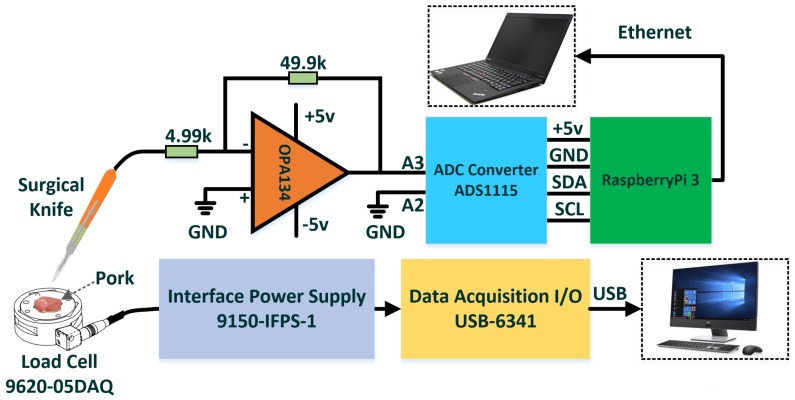
Schematic diagram of surgical knife.

Figure 2 shows the measurement setup that is comprised of the operational amplifier (OPA134, Texas Instruments, Dallas, TX, USA), analog-to-digital converter (ADC) (ADS1115, Texas Instruments) and micro-computer (RaspberryPi 3, Raspberry Pi Foundation, Cambridge, UK). The operational amplifier is constructed by using the junction field-effect transistor (JFET), in which the electrons flow through with two ohmic electrical connections (drain and source ports).

The 4.99 and 49.9 K resistors are used to construct the differential amplifier with a gain of 10. The power supply (Kikusui PMM25-1TR, KIKUSUI ELECTRONICS CORP., Kanagawa, Japan) of the operational amplifier is ±5 V with electrical noise. A 16-bit ADC is utilized to convert the analog signals to digital signals of the collected signals from the surgical knife. The inner-integrated-circuit ($I^{2}C$) protocol is used for communication between the ADC and micro-computer with serial data line (SDA) and serial clock line (SCL) ports. The power supply of the ADC is from a +5 V pin of the micro-computer that is also used for the signal acquisition and post-processing of the surgical knife with the Python programming language. The laptop serves as the display of the micro-computer that is connected by an Ethernet cable. Meanwhile, the signals from the high-precision load cell or force transducer (F/T Sensor Gamma, ATI Industrial Automation, Apex, NC, USA) are used as the reference of the proposed perceptual surgical knife. The load cell is a tri-axis transducer for measuring the force applied to the human body. The interface power (IFP) supply (9105-IFPS-1, ATI Industrial Automation) is used to power the load cell, and the data acquisition (DAQ) card (USB-6341, National Instruments, Austin, TX, USA) is used to communicate with the load cell.

### 2.3. Signal Processing of Perceptual Surgical Knife

A micro-computer with a Linux system is used for the signal processing of the perceptual surgical knife. For the DAQ, the differential analog signals from ports A2 and A3 of the ADC (refer to Figure 2) are collected with the Adafruit ADS1X15 Python library in the micro-computer. The sample rate of the ADC is 860 samples per second with a programmable gain amplifier to control the range of analog inputs. The full-scale range of the ADC is ±4.0965 V in this application, and the scale factor of the ADC is $1.25\times 10^{-4}$ ($4.096/2^{15}-1$). The high-frequency noise of the power supply from the micro-computer degrades the quality of received signals. It is difficult to distinguish between low-frequency motions, such as tapping, sliding or cutting of the perceptual surgical knife, and the received signals disturbed by high-frequency noise; thus the wavelet denoising method is proposed to filter the high-frequency noise. Wavelet transformation uses long-term intervals to extract precise low-frequency components and seek high-frequency signals by narrowing windows [18]. In wavelet transformation, the mother wavelet generates building blocks to represent a general function at different scales and positions. The discrete wavelet transform of the signal $x\left(t\right)\{x\left(t\right)\in L^{2}\left(R\right)\}$ can be expressed by the following expression [19]:

(2) $$x\left(t\right)=\sum_{n\in Z}C_{J,n}\phi_{J,n}\left(t\right)+\sum_{j=J}^{\infty }\sum_{n\in Z}d_{j,n}\psi_{j,n}\left(t\right)$$

where $\phi_{J,n}\left(t\right)$ is the scaling function and $\psi_{j,n}\left(t\right)$ is the mother wavelet function. *J* is the initial resolution; $c_{j,n}$ and $d_{j,n}$ are the scaling coefficients and wavelet coefficients, respectively, which have different expressions for different wavelet transformations. In this paper, the maximal overlap discrete wavelet transform (MODWT) is selected as the wavelet filter, and the scaling coefficients and wavelet coefficients are defined by the following equations [20,21]:

(3) $$\begin{array}{ccc} c_{j,n} & = & \sum_{l=0}^{L-1}\frac{1}{\sqrt{2}}g_{l}c_{j-1,(n-2^{j-1}l)\;mod\;N} \end{array}$$

(4) $$\begin{array}{ccc} d_{j,n} & = & \sum_{l=0}^{L-1}\frac{1}{\sqrt{2}}h_{l}c_{j-1,(n-2^{j-1}l)\;mod\;N} \end{array}$$

where $g_{l}$ and $h_{l}$ denote the coefficients of the scaling filter and wavelet filter, respectively; *L* is the length of the wavelet filter; n=0,1,2,⋯,N−1 and *N* is the length of the time series. To demonstrate the functionality of perceptual surgical knife with the MODWT denoising method, the index finger of a healthy man was used for the test. Table 1 gives the results of the wavelet denoising. The maximum (minimum) voltages of the original signals and wavelet denoising signals were 156 mV (−74 mV) and 54 mV (0.7474 mV), respectively. Additionally, the standard deviation (std) decreased from 0.0031 to 7.6959 $\times \;10^{-4}$ by using the wavelet denoising method, but the mean did not change (3.6 mV).

**Figure 3:**
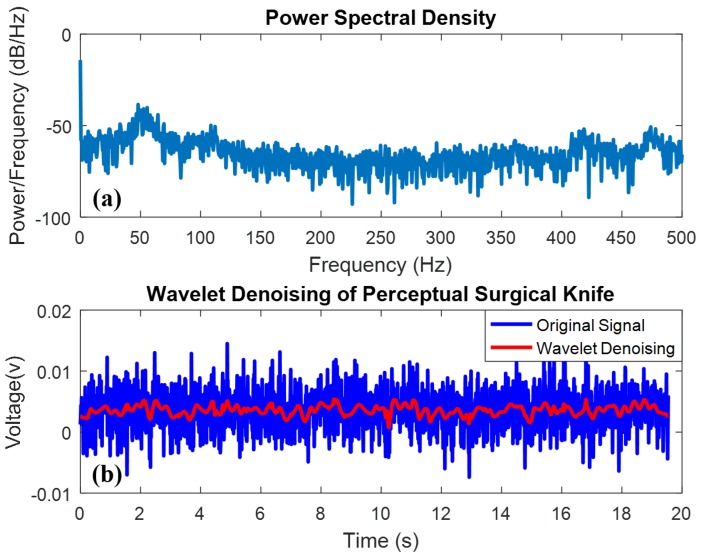
Wavelet denoising of perceptual surgical knife. (**a**) Power spectral density of the high-frequency noise; (**b**) wavelet denoising of perceptual surgical knife.

Figure 3b shows the collected signals from the index finger. The voltages of the received signals ranged from −74 to 156 mV and were disrupted by the high-frequency noise from the power supply of the micro-computer, as explained by the power spectral density in Figure 3a. The result of the power spectral density was obtained by the Fourier transform method. The result of the MODTW denoising approach indicates that the wavelet denoising method can effectively remove high-frequency noise.

## 3. Results and Discussion

**Figure 4:**
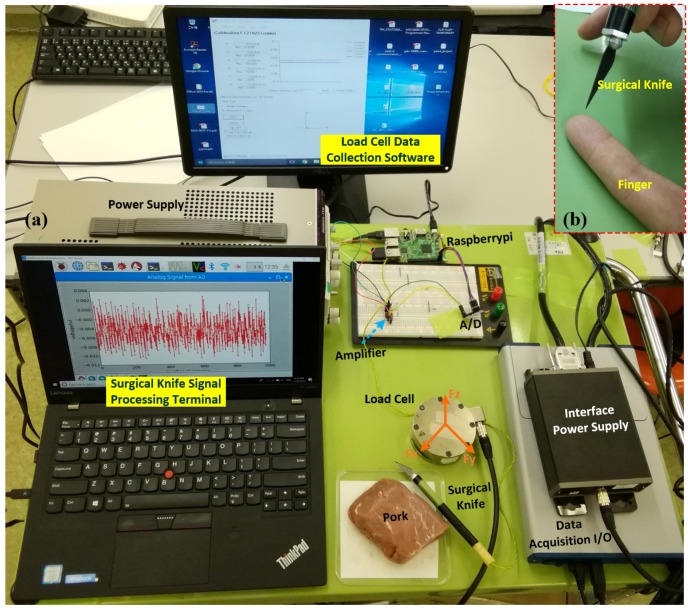
Experimental setup of wavelet-denoising-based perceptual surgical knife. (**a**) Experimental setup; (**b**) experimental example. $F_{x}$, $F_{y}$ and $F_{z}$ represent the output forces of load cell along *x*-axis, *y*-axis and *z*-axis, respectively. The sampling rate of the surgical knife was 860 samples per second, and the sampling rate of the load cell was 1000 samples per second with a 16 sample average approximation.

Three experiments were conducted to demonstrate the capability of the proposed perceptual surgical knife. In the first experiment, the index finger was used to test the tapping and sliding motions of the surgical knife. Figure 4a shows the experimental setup built according to the schematic diagram of the measurement setup (refer to Figure 2). The experimental example is shown in Figure 4b.

**Figure 5:**
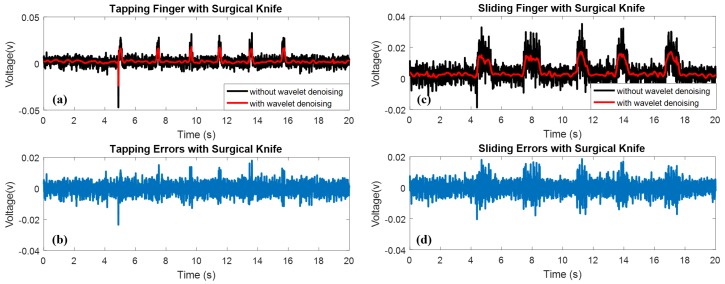
Experimental results of surgical knife with tapping and sliding motion. (**a**) Tapping results of surgical knife with wavelet denoising; (**b**) noise in the tapping experiment; (**c**) sliding results of surgical knife with wavelet denoising; (**d**) noise in the sliding experiment.

Figure 5 shows the experimental results of the perceptual surgical knife with tapping and sliding motions to detect the electrons in the human body. In the tapping experiment, the surgical knife tapped the index finger every 2 s, and the motion lasted for about 0.2 s for each tap. The received signals from the surgical knife were noisy compared to the signals with the wavelet denoising approach, and six tapping peaks were distinguished in the results of the tapping experiment with the wavelet denoising method (refer to Figure 5a).

**Figure 6:**
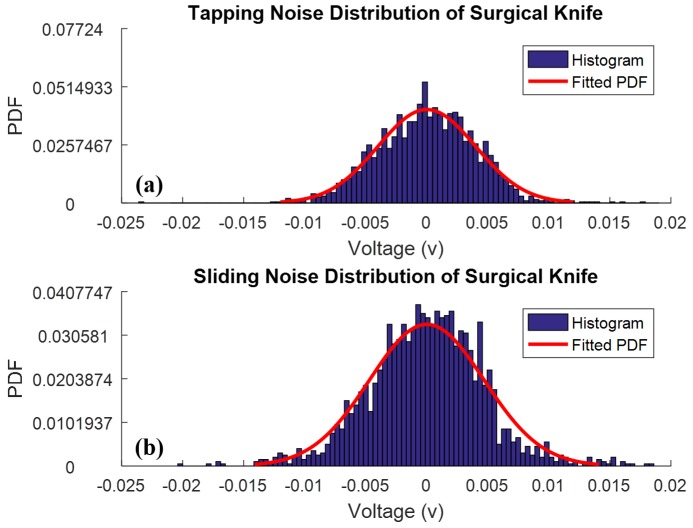
Noise distribution of detected signals from surgical knife. (**a**) Tapping noise distribution of surgical knife; (**b**) sliding noise distribution of surgical knife.

Similarly, the perceptual surgical knife slid on the index finger every 2 s, and the motion lasted for approximately 0.4 s for each slide. Five peaks that indicate the sliding motion were observed; however, the peaks are submerged in the noise. With the wavelet denoising approach, Figure 5c shows five visible sliding peaks. Figure 5b,d illustrates the noise of the surgical knife with tapping and sliding motions, respectively. Figure 6a,b shows the probability density functions (PDFs) of the tapping and sliding noise that are characterized by a zero mean and a Gaussian distribution.

These experimental results indicate that the perceptual surgical knife achieves good functionality in detecting tapping and sliding motions. In addition, the wavelet denoising method enables us to remove the Gaussian noise that exists in the perceptual surgical knife.

**Figure 7:**
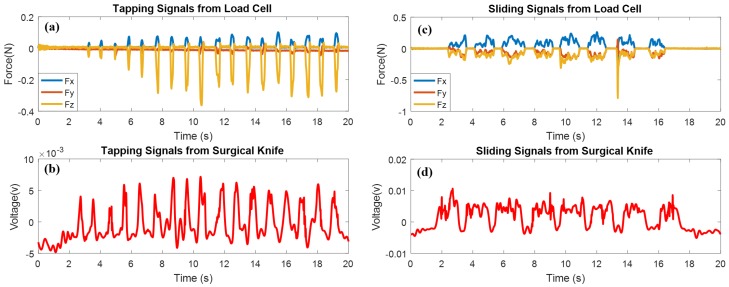
Tapping and sliding experiments of perceptual surgical knife. (**a**) Tapping results of load cell; (**b**) tapping results of surgical knife; (**c**) sliding results of load cell; (**d**) sliding results of surgical knife.

The experimental results in Figure 5 demonstrate the functionalities of the perceptual surgical knife (tapping and sliding). To strengthen the experimental reliability, the second experiment was designed with a high-precision load cell as the reference. In addition, we chose a piece of pork as the alternative to the index finger, because the tiny motion of the finger introduces interference to the sensing signals of the surgical knife. Figure 7a shows the tapping results of the surgical knife from the load cell. The entire experiment ran over 20 s, and the time interval for each tap was 1 s.

Eighteen tapping motions were detected by the 3-axis load cell whose *z*-axis is sensitive to the load force. Seventeen periodic peaks with different amplitudes were detected by the surgical knife, as the first tapping force (0.05565 N) was insufficient for the surgical knife to detect (refer to Figure 7b). However, the second tap of just 0.00925 N more than the first tap was detected by the surgical knife. These results indicate that the measurement limit of the surgical knife is 0.0649 N, which validates the high sensitivity of the perceptual surgical knife.

Another sliding experiment was designed to demonstrate the performance of the surgical knife further. To retain the consistency, the experiment was restrained to 20 s, and the interval of sliding was 0.5 s for each motion. Apparently, eight sliding motions appeared in the results of the load cell (refer to Figure 7c). Initial sliding motions of the surgical knife showed the same phenomenon as the tapping motions, because each sliding motion began as a tapping motion in the moment of contact with the pork. Therefore, a peak appeared in the initial detecting signals of the sliding motions. However, once the sliding began on the pork, the intensity of the sensing voltage gradually reduced. This was consistent with the measurements obtained from the load cell; the highest values of the coefficient of friction (COF) were found at the beginning of the running-in periods, and then the dynamic friction decreased to a stable value. Figure 7d gives the sliding perceptual result of the surgical knife.

**Figure 8:**
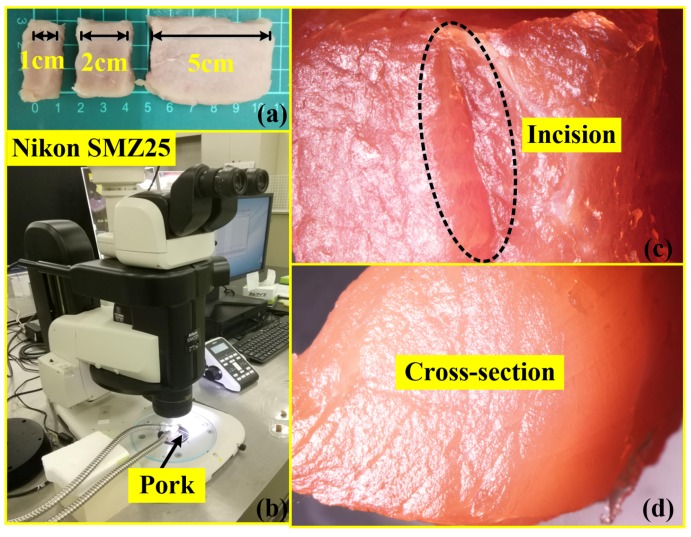
Samples for cutting experiment. (**a**) Three samples of different dimensions; (**b**) Nikon’s SMZ25 stereomicroscope (Nikon Instruments, Tokyo, Japan); (**c**) pork incision; (**d**) cross-section of cutting.

In the third experiment, three samples with precise dimensions were used for demonstrating the cutting function of the perceptual surgical knife. As shown in Figure 8a, the dimensions of the samples were 1 cm × 3 cm × 0.5 cm, 2 cm × 3 cm × 0.5 cm, and 5 cm × 3 cm × 0.5 cm (length, width, and height, respectively). The surgical knife cut each sample three times in the horizontal direction; simultaneously, the load cell under the samples was used for the measurement of the load force impacting on the samples. In addition, Nikon’s SMZ25 stereomicroscope (Nikon Instruments, Tokyo, Japan) (refer to Figure 8b) was used for capturing the incision images of the port (refer to Figure 8c) that was cut by the perceptual surgical knife. Figure 8d shows the cut-off cross-section of the pork sample.

**Figure 9:**
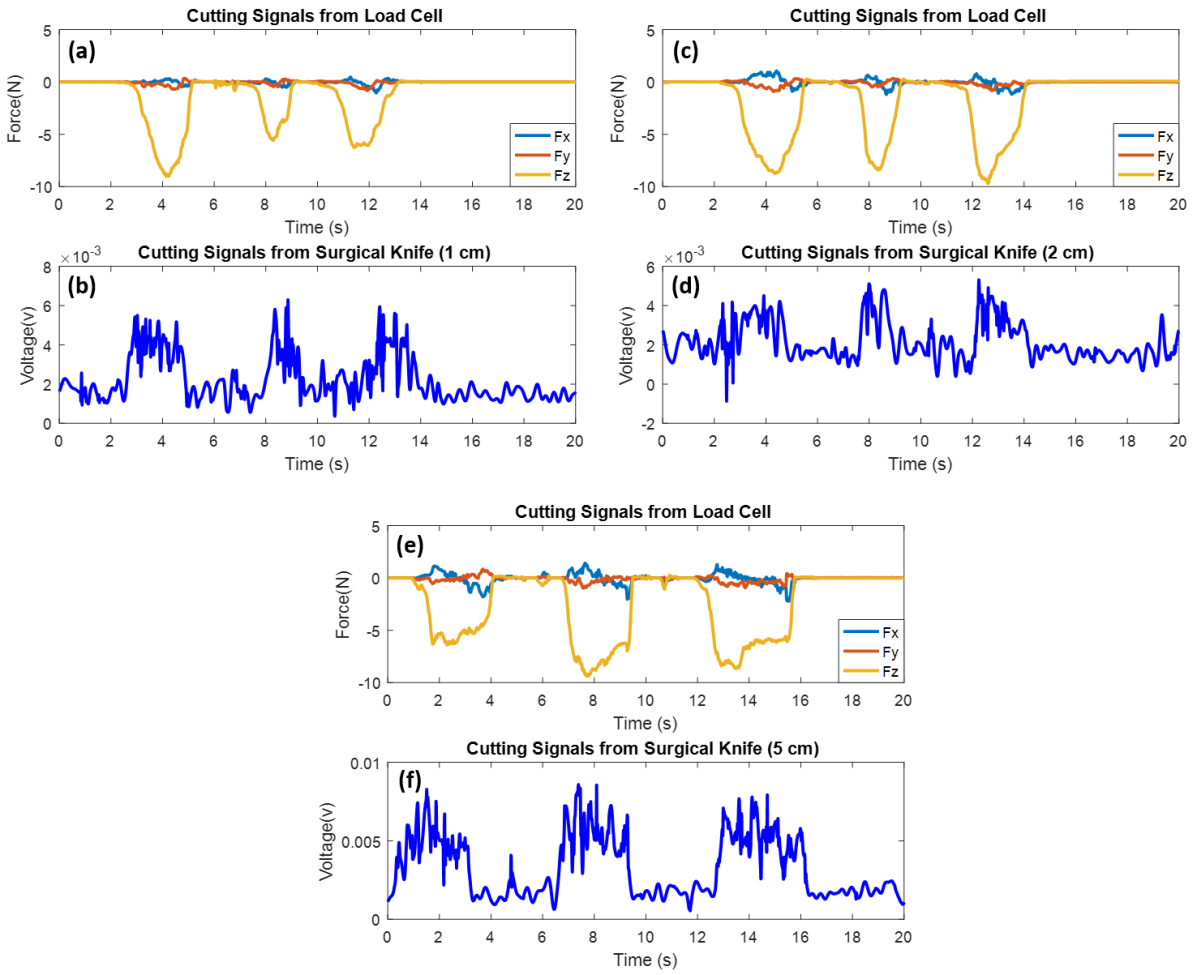
Perceptual cutting signals from surgical knife with (**b**) 1 cm sample, (**d**) 2 cm sample, and (**f**) 5 cm sample. Cutting load force from load cell with (**a**) 1 cm sample, (**c**) 2 cm sample, and (**e**) 5 cm sample.

Figure 9 shows the cutting experimental results with the proposed perceptual surgical knife. As shown in Figure 9a,b, the surgical knife cut the sample with dimensions of 1 cm × 3 cm × 0.5 cm. The repeatability was evaluated by cutting each sample three times. In particular, the maximum load forces when cutting the first sample (1 cm × 3 cm × 0.5 cm) were 8.934, 5.585, and 6.275 N; concurrently, the average perceptual peak voltages measured by the surgical knife were 0.0039, 0.0037, and 0.0036 V with std’s of 0.0007, 0.001, and 0.001, respectively. The results show that the perceptual signals of the surgical knife exactly matched the measurements of the load cell in the time frame, which indicates that the surgical knife has good perceptual ability and good repeatability. Two other experiments were carried out to provide a comparison of different cutting lengths by using 2 and 5 cm length samples, as reported in Figure 9c–f. Similarly, the cutting peaks were also observed in both experiments. Table 2 presents the results of these two experiments.

## 4. Conclusions

In summary, the proposed perceptual surgical knife has the capability of detecting physical motions such as tapping, sliding and cutting by detecting the electrons or electric field of the human body. The wavelet denoising method for the signal processing of the surgical knife is able to eliminate the high-frequency Gaussian white noise of perceptual signals. Tapping, sliding and cutting experiments with both a human index finger and pork (the alternative to the index finger) were conducted to demonstrate the perceptual ability of the surgical knife. The experimental results show that the tapping and sliding motions in the experiments were distinguishable under the light-touch regime. Moreover, the cutting perceptual signals of the surgical knife were consistent within the time frame. Therefore, the perceptual surgical knife is characterized by a high sensitivity and high repeatability, which make it highly suitable for robotic surgery.

## Figures and Tables

**Table 1:** Results of wavelet denoising.

Parameters	Maximium	Minimum	Mean	Standard Deviation
Original signal	156 mV	−74 mV	3.6 mV	0.036
Wavelet denoising	54 mV	0.7474 mV	3.6 mV	7.6959 $\times \;10^{-4}$

**Table 2:** Results of cutting experiments with different samples.

Sample Length	Cutting Number	Load Force	Perceptual Voltages (Average)	Standard Deviation
2 cm	1	8.747 N	0.003 V	0.0009
	2	8.378 N	0.0037 V	0.00085
	3	9.707 N	0.0034 V	0.00069
5 cm	1	6.358 N	0.0049 V	0.0011
	2	9.358 N	0.0055 V	0.0013
	3	8.659 N	0.0052 V	0.0011
